# Knowing and accepting oneself: Exploring possibilities of self-awareness among working autistic young adults

**DOI:** 10.1177/13623613221137428

**Published:** 2022-11-21

**Authors:** Hanna Bertilsdotter Rosqvist, Lill Hultman, Johan Hallqvist

**Affiliations:** Södertörn University, Sweden

**Keywords:** adults, autism, autistic-centred support, neurodiversity, self-awareness

## Abstract

Autistic people have historically been described as incapable of developing a deeper sense of self-awareness, and autistic understandings of self-awareness have been largely disregarded. The aim of this study is to explore the way young autistic adults try to understand their functionality and who they are, or to develop their sense of self-awareness, in work and in private life contexts. In 12 qualitative interviews conducted with four autistic adults without learning difficulties, we identified a rich set of reflections on knowing and accepting oneself. The overarching theme of self-knowledge has three subthemes: learning from previous experiences, learning about oneself by securing the support of others, and understanding and accepting autistic functionality. The strategy of self-knowledge was used by these young adults to help them achieve functional lives in the work and private domains. Our results show that young autistic adults both actively explore and develop their self-awareness. We suggest that it is important for practitioners and employers working with autistic individuals to engage with their journeys of self-awareness as a vital part of understanding and supporting them.

**Lay abstract:**

When researchers and professionals talk about autism, they commonly point out problems and risks with autism or being autistic. Several interventions are based on the idea of the problems and risks of autism. Another way of talking about autism is to point out autistic people’s strengths and strategies which they use to handle barriers and problems in their lives in order to live good lives on their own terms. In this article, the researchers explore how autistic young adults formulate their own difficulties, strengths and support needs in order to get right support from support people. To be able to formulate this, autistic people need to get to know oneself and one’s own way of functioning. Autistic own self-knowledge must be central when formal support people, such as social workers, formulate support and interventions aimed at helping autistic people, in order for the support/intervention to be helpful.

## Introduction

This article explores self-awareness among working autistic young adults. The focus is on their strategies for knowing and accepting themselves in order to achieve functional working and private lives. The issue of autistic self-awareness has generally been approached from a deficit lens. However, more recent research has challenged this by stressing the possibilities and importance of autistic self-awareness. We extend this shift in perspective to vocational training, arguing that autistic adults see self-awareness as a pivotal component of both their vocational and their private lives.

There is a general agreement that self-awareness is foundational to conscious experience (Lou et al., 2017). However, autistic people have been described as lacking in self-awareness and the ability to differentiate the self from the non-self (Ferrari & Matthews, 1983). This idea is based on neurotypical developmental norms regarding self-recognition (Ferrari & Matthews, 1983) and self-object differentiation (Pallanti & Quercioli, 2000), as well as expectations of differentiating the self from others in neurotypical ways (Hobson, 2006). These include facial emotion recognition and expression (Baltazar & Conty, 2016), and expressing feelings within social-relational and emotional domains of self-awareness (Hobson, 2006).

Scholars have suggested that impairments in social communication among autistic people might be caused by deficits in ‘perspective-taking, language processing, and self-other representation’ (Kana et al., 2017, p. 418). For example, Mundy et al. (2010) propose that ‘an early, chronic disturbance in the capacity for integrating self-and other referenced information may have cascading effects on the development of self-awareness in autism’ (p. 408). However, other researchers have argued that there is no significant difference between autistics and neurotypicals regarding objective or subjective self-awareness (Lind et al., 2019).

Autistic people have also been associated with different memory processes connected to the self in comparison with neurotypicals (Bowler & Gaigg, 2008). For example, Bowler and Gaigg (2008) have noted a limited sense of self-awareness when evoking one’s experienced past and encoded context among autistic people. However, autobiographies written by autistic people have challenged assumptions of autistic people’s autobiographical memory deficits, as writing autobiographies requires abilities such as self-awareness, imagination, competence and a will to communicate (Young, 2012).

Stout (2019) argues that autism must be understood as a disarrangement of emotional self-awareness, stressing that autistic people seem to be detached from their emotions. However, several researchers question this understanding of autism, stressing how autistic people might process and adopt internal and external information differently from neurotypical people (Failla et al., 2020), and that autistic individuals’ self-awareness is not diminished but possibly harmed by their negative self-beliefs due to low self-belief in their socio-emotional competence (Huggins et al., 2021). For example, Sumiya et al. (2018) show that autistic adolescents have a strong self-awareness of their social challenges and make conscious attempts to manage these challenges.

Stenning (2020) argues that research describing autistic people as lacking in or having limited self-awareness or difficulties with recognizing internal states can be understood as one strand of common depictions of autism as a deficit. Such depictions dehumanize autistic people by denying them characteristics such as introspection and a developed sense of self. McDermott (2022) argues that autistic individuals may experience difficulties in recognizing internal states due to the mismatch between what is felt (one’s own internal states) and what one is expected to feel according to neurotypical or what McDermott refers to as ‘neuroconventional’ norms. In contrast to research suggesting that autistic people have a limited or impaired sense of self, Lilley et al. (2021, p. 1395) note, from the results of interviews with late-diagnosed autistic adults, that interviewees ‘demonstrated a deep capacity for self-reflection, highlighting the variability of autistic lives and the socio-historical contexts that shape individual biographies, including experiences of stigma and discrimination as well as the empowering potential of identifying as autistic’ (see also Crane et al., 2019).

Researchers stress self-awareness as a key to autistic people’s flourishing. Müller et al. (2008) show how autistic adults develop a greater self-awareness by relying on external support from others, communication support and self-initiated strategies to manage social anxiety. Autistic self-awareness and personal control are associated with protection against autistic burnout (Mantzalas et al., 2022). Developing autistic self-awareness is pivotal to recognizing and supporting passions and interests, cultivating relationships with others and developing strategies to navigate the environment (South & Sunderland, 2022).

Self-awareness is also central to social and vocational opportunities, which can capitalize on strengths and interests (South & Sunderland, 2022), leading to the development of expert competence at work (Webster & Garvis, 2020). Webster and Garvis (2020) identify four key factors among young autistic men who regard themselves as successful at work: being oneself, being a competent professional, overcoming problems in a neurotypical world, and connecting and relating to others. Successful autistic people can provide ‘a source of wisdom’ for other autistic individuals, families and human services workers (First et al., 2019; South & Sunderland, 2022).

Closely connected to an autistic self and self-reflexivity are a range of interventions aimed at increasing autistic people’s self-awareness. Vocational rehabilitation interventions (VRIs) aim to support autistic people to gain and maintain employment in the jobs market, such as targeted job placements (Kaya et al., 2018). VRIs have shown positive results assisting young autistic adults to expand their capacities for independent self-care, maintain employment and increase their working hours and independence at work (Wehman et al., 2017). However, several researchers have pointed out that environmental factors can be both barriers to and facilitators of the employment of autistic people, and the critical need for interventions that target contextual factors if employment outcomes are to be improved (Scott et al., 2019).

The aim of this study is to explore young autistic adults’ own ways of trying to understand their functionality and who they are – or, in other words, to develop their sense of self-awareness – in working and private life contexts. To clarify the aim, three research questions were formulated:

- What are the possibilities for young autistic adults to explore their sense of self-awareness?
- What is the importance of developing self-awareness?
- How do working autistic adults gain and use self-knowledge in order to improve their working and daily lives?

## Method

Engaging with autistic people’s own perspective of their selves and their understandings of other’s perceptions of them is central to exploring autistic self-awareness (Huang et al., 2017). We adopt an autistic-centred approach to research and a neurodiversity approach to autism. An autistic-centred approach to research first and foremost refers to research agendas and questions formulated by autistic people and the autistic community (see Roche et al., 2021), as well as the importance of listening to autistic people’s own perspectives and experiences of autism (Rapp et al., 2020) and highlighting autistic people as agents in their own lives (First et al., 2019). In addition, it refers to research methods that actively enable autistic functionality and ways of communication. A neurodiversity approach to autism refers to how autistic individuals can be ‘differently neurologically wired’ (Singer, 2016), as well as how research about autism tends to be framed by neuroconventional norms (Bertilsdotter Rosqvist, Botha, et al., 2022), focussed on deficit-oriented approaches (Cribb et al., 2019). A neurodiversity approach strives to overcome these deficit-oriented stereotypes by adopting more positive and nuanced views of autistic lives. This research is autist-led. The first author is autistic and the whole research group is neuro-mixed, consisting of researchers who identify as either neurodivergent or neurotypical. The research questions, theories used and interviewing style were informed by the first author’s engagement in the autistic community.

### Participants

This study is part of a larger research project with the overall objective of exploring different aspects of experiences of supported employment among young autistic adults and young adults with a learning difficulty. The whole data set consists of 16 in-depth interviews with five men and three women, recruited with the support of coaches at a vocational service intervention centre in Stockholm. The participants are in their 20s or 30s, except for one who is in his 40s. Two of the women have a learning difficulty. The other interviewees are autistic with no learning difficulty. All the participants were living in different suburbs or municipalities surrounding Stockholm city at the time of the interviews. The interviewees had both middle-class and working-class backgrounds in relation to higher education. Six of the interviewees were born in Sweden and two were born abroad but migrated to Sweden in their early childhood.

For this study, 12 in-depth interviews were conducted with four young autistic adults without a learning difficulty. All the names are pseudonyms:

1. Alice, in her 20s, currently in a VRI where together with a coach she is mapping out her interests and strengths in order to match these with a potential employer’s needs (three individual interviews).
2. Hugo, in his 20s, is employed in the IT sector (three individual interviews).
3. Lucas, in his 20s, has a managerial position that involves customer responsibility (three individual interviews).
4. William, in his early 40s, works as an archivist (interviewed three times, twice in a pair and once individually).

### Data collection

Initially, the first author met up with the coaches and presented the project to them. The coaches were invited to ask questions and provide input on the research design, and were asked to help recruit interviewees. The coaches asked clients they had been working with and others interested in participating in the study. For the first interview, the coaches ensured that the interviewees found their way to and arrived on time for their interview. For the following interviews, the first author took responsibility for supporting participation by sending text message reminders. The interviewees decided whether they wanted to take part in individual or paired interviews. Among those interested in paired interviews, the coaches who knew the interviewees suggested which interviewees should be paired together. Due to difficulties in finding a time that suited the interviewees who preferred to be interviewed together, some paired interviews were transformed into individual interviews.

As a crucial part of an autistic-led research process, the autistic interviewer’s own experiences with autistic communication and sociality were central (Bertilsdotter Rosqvist, 2019). This can be contrasted with the challenges of cross-neurotype communication (Hillary, 2020) and the risks of double empathy problems (Milton, 2012) in an interview setting where the interviewer is non-autistic and the interviewee is autistic.

During the interviews, the conversation quickly adopted a certain pace that allowed for processing time. Direct eye contact was limited in order not to disturb processing by either the interviewer or the interviewee(s). Rather than follow a structured interview guide and force the interviewee(s) to adapt to a predefined structure, the interviewer used open-ended questions that followed the interviewees’ interests and ways of narrating, albeit steering the interview to keep within the areas of the research topics of working life, private life and support. The interviewer included visual communicative support, such as writing on a whiteboard where she further developed and explained her questions and the associations she had in relation to the interviewees’ narratives. The interviewer did not explicitly disclose being autistic herself but did not try to pass as non-autistic and did her best to communicate in an ‘autistic affirmative’ way. Her communicative style was a way of disclosing implicitly and ‘normalizing’ an autistic way of being rather than othering it, which she felt made the interviewees more relaxed.

The interviews lasted for between 30 min and 2 h and were conducted in Swedish by the first author. The interviews covered experiences and expectations of working life in the ordinary labour market, experiences beyond the workplace and support, including self-support strategies. The interviewees were interviewed one to three times, in pairs or individually at the office of the vocational rehabilitation service provider. The original study was approved by the Ethics Committee of Regional Ethical Review Board in Umeå, Sweden [2017/43-31].

### Analysis

Qualitative inductive content analysis was used to analyse the data (Elo & Kyngäs, 2008). The interviews from the whole data set were read by the authors several times to obtain an overview of the material. Four general themes emerged: functionality, support and strategies, learning and self-awareness. For this article, data about self-awareness were analysed, drawing on interviews with four autistic young adults. The coding process was influenced by classical grounded theory coding, using the constant comparative method and memo writing. The authors performed individual line-by-line coding separately, followed by discussions and comparisons in relation to the emerging categories (Glaser & Strauss, 1967). Similar categories were merged into common categories. The findings present the overall theme and its constituent categories. The categories are illustrated by quotes and examples from the data.

## Findings

The young autistic adults in this study are involved in trying to understand and learn who they are and how they function in a variety of social interaction contexts, in both work and leisure time. They are explicitly engaged in developing relationship and problem-solving skills. We identified this overall strategy as *Knowing and Accepting oneself*. This strategy was made up of three substrategies: *learning from previous experiences*, *learning about oneself by securing support from others* and *understanding and accepting autistic functionality*.

### Learning from previous experiences

At work, it is important for employees to engage in dialogue with their work manager and to be open about when and under what circumstances they might benefit from external support. Managers are often described as not understanding the consequences of the employees’ functionality, resulting in unrealistic expectations of their work performance. The employer might assume that an autistic person understands work-related tasks and concepts that autistic functionality make difficult to grasp, or may assume that an autistic person has the ability to perform tasks in the same way as a neurotypical person. Receiving the right kind of support requires mutual respect, responsibility and cooperation between the employee and employer. The employee needs to develop self-awareness and an ability to communicate needs. At the same time, employers or managers must be willing to understand, listen empathetically and adjust according to the support needs communicated by autistic employees.

Participants told us that previous experiences of similar work tasks made it easier for them to adapt and cope with their work:

Before I started [this] job, I knew quite a lot about what the job entailed. That you should pick up papers, archive and delete stuff that should not be at work. You should be vigilant about what you do, so it doesn’t go wrong. It’s better to be too careful than not [careful enough]. So that you do exactly the right thing. You had to sort [papers in the archive] in a structured manner, otherwise it will be completely wrong. At first, I thought that it was very difficult, but it wasn’t very difficult. You learn that [kind of] stuff pretty quickly . . . It’s all about being very careful and knowing what you’re doing. (William)

Participants described adjusting leisure activities according to endurance and intensity, providing insights into how much energy it takes to participate in different types of activities, as well as how much time needs to be spent recuperating afterwards. Learning from previous experience, Hugo tries to avoid situations that make him feel emotionally unbalanced:

I’ve learned to try not to plan too many activities in the same week. I try to schedule things like doctor’s appointments and things like that. I try to be careful that I don’t do anything else that week then. Because I know it’ll take a lot [of energy]. I don’t know, but many [people] seem to be able to do several things without problems. It’s incomprehensible to me that people can handle it, it doesn’t work for me. I’ve noticed that it doesn’t. (Hugo)

For some of the young adults, socializing with friends takes a lot of energy. They told us that it can be difficult to know how much time they need to spend alone compared to time spent with others. It can be particularly difficult if it varies from week to week:

I have no ordinary weeks. I am very special as a person so sometimes it can be like, I can’t meet anyone this week, then I don’t hang out with anyone, and next week I have to go out and have coffee with friends every night. So, it would be very difficult to have an overall norm. (Lucas)

Participants stressed that socializing with a group of people usually takes more energy than socializing with one person. These young adults spoke about planning both the duration of activities and the time needed for recuperation after engaging in social activities: ‘When it’s a larger group that you should be part of and are expected to be an active part of it . . . I still know how, I’m not impossible in such situations, but I get very tired’ (Hugo).

There must also be a balance between alone time and time spent with others. It is therefore helpful to plan time for being alone:

To be able to find that little spot for oneself some time every week when one has the chance to do whatever one wants to do, being alone without having anybody to disturb you, to turn off your brain, not having to think; not having to think or do, just being. (Lucas)

Identifying, accepting and expressing individual needs in relation to social interaction is an important step to expressing the need for social withdrawal. This was identified by the participants as an important coping strategy.

### Learning about oneself through securing support from others

Participants told us that securing support from others was linked to learning about oneself and one’s capacities, which entails finding strategies to cope with challenges. Support was variously described as involving close relationships with parents, partners or friends who provide advice or necessary practical support in daily life, specifically when these young adults struggle to manage on their own. The need for support was often raised in connection with the transition to independent living, when the participants reported being expected to take responsibility for managing their own household, including tasks such as buying food, paying bills, washing clothes and cleaning.

With advanced planning and access to informal support, the participants tried to ensure that they could handle everyday chores. William, who moved to his own apartment when he was 30 years old and has lived independently for approximately 10 years, mostly manages the household by himself. Nonetheless, he knows that his parents will help him when necessary. William has learned how to resolve recurring tasks by creating routines

My mother comes around and does a thorough clean once a year, but I can do most things myself . . .// It isn’t easy to live away from [your parents’] home, it’s a little tricky in everyday life. I’ve put most of my important bills on direct debit . . .// So, I don’t even have to think about it.

When it comes to cooking, William has learned how to make a limited number of dishes by himself; and if he wants to do more advanced cooking, he turns it into a social activity by cooking with friends, which makes it ‘more fun to cook’.

Hugo compares himself with others and wants to fit into what he perceives as part of an adult lifestyle. Although he wants to work part-time, he knows that this is not a realistic goal, since he already has difficulties with coping with the balancing act between work and spare time:

Right now, I work three days a week, four hours each day . . . 30 per cent, or 33 per cent, or something . . . my aim is to get up to 50 per cent. I’ve worked 50 per cent at some point, but then I still lived at home [with my parents]. I have a very hard time finding the energy to do things at home. I have to ignore it to cope [with work]. It’s hard to come up with more [time] than what I’m working now. (Hugo)

Thus, Hugo’s ability to work is connected to both self-management skills, which includes self-awareness of his strengths and weaknesses, and practical and emotional parental support. Hugo’s challenges lie in finding a balance between work and leisure, where the number of hours he can cope with working each day is linked to the support he is receiving from his parents. However, he worries about his life when his parents get older or die:

I know I’ll find it more difficult when my parents get older and disappear because I’m pretty dependent on them in many ways. I think I’ll probably get quite lonely. [. . .] I expect that I won’t find it so easy in the future. (Hugo)

Hugo is concerned about how his future will unfold since his parents play a crucial role in providing both social and emotional support and he cannot imagine a fulfilling life without the support of his parents.

### Understanding and accepting autistic functionality

Our participants talked about how it takes time to know oneself. Self-awareness often comes through experience-based learning, in which these young autistic adults discover how they function and their likes and dislikes – both at work and in leisure time:

I spent a very long time getting to know myself – ‘how I feel about doing this, what I think about that’, and how it’s important to find a balance in what you like to do and what you don’t like to do. (Lucas)

For Lucas, self-awareness entails the capacity to understand what he needs to do to be able to take responsibility for his emotional well-being, such as the capacity to care for himself and being able to take responsibility for his actions. He reflects on his own behaviour and analyses the outcome:

I ask myself philosophical questions. It allows you to reflect on what you do. ‘Do I feel good when I do this? No’; ‘Is it necessary to do this or do I enjoy doing it?’ Or it makes me realize that, in this situation I reacted . . . so what do I have to do to avoid doing this or to avoid the situation? How can I improve this situation? (Lucas)

The young adults interviewed underlined the importance of not making general assumptions about autistic functionality. Instead, they suggested that it is crucial to find out what applies to each individual and to make appropriate adaptations for that person:

Some parts you can recognize but, as I said, Asperger’s varies from person to person, so you notice some are very social, others are very shy, and some are very afraid of conflict. . . . There are some who can’t work and some who have problems with this and that. Autism and Asperger’s are very wide [concepts] so it’s very difficult to find someone with whom you have a lot in common; it’s just pure luck really. (William)

For some of the young autistic interviewees, receiving a diagnosis worked as a source of understanding and finding strategies to handle the challenges related to it:

I didn’t receive my diagnosis until about 11 months ago. For a long time, I didn’t understand myself or the way I function, for example, how I could function and have a lot of energy for one to two weeks and then suddenly not be able to do anything. Now I’m starting to understand a little bit more about how I work . . .. It feels like I’m starting to realize that I need to kind of listen to myself. (Alice)

However, receiving a diagnosis as an adult can be difficult to process. On the one hand, it can be a relief to be able to understand that there are common characteristics among autistic people that can be understood and shared by others. On the other hand, it can raise questions about identity and competence:

I’ve probably always had difficulties coming to terms with certain parts of the diagnosis, but at the same time it was nice when I got it because it provided answers to some things – why I had such a hard time with certain things. (Hugo)

Since non-autistic people do not always notice or understand that a person is autistic, it can be a challenge to get non-autistic people to adapt conditions at work for autistic people. Hence, a great deal of responsibility is placed on the autistic person:

If you’re very high functioning, as I am, people probably have a hard time understanding that I have difficulties with certain things. It’s not really apparent in the situations where I meet people; it’s more noticeable at home. I think the expectations of those around me are different when it doesn’t appear that I have any problems. (Hugo)

Our participants pointed out that it is important for employers to show a genuine interest in understanding how autistic functionality works for the individual autistic employee. Otherwise, it is easy to stereotype a person’s autism based on a more general description or understanding of the autism diagnosis, or to have unrealistic expectations of the autistic employee:

For some [autistic people] it’s like having to have very strict routines and wanting to do the same thing all the time. Some [autistic people] are much more chaotic [than others] and I’m more like that. It works better if I do different things. I have a very hard time with doing the same thing. However, I’m very good at focusing, because that’s typical of us . . . Asperger stuff, I guess . . . that you’re very good at focusing on a task and doing it very well; and I have always been good at that. But I don’t want to always do the same task just because of that. Because then I get under-stimulated in some way and I easily give up if I feel that what I am doing is pointless. (Hugo)

For Alice, when she talks about feeling like a failure, she means prior to the diagnosis – she is now having to unlearn this negative self-understanding in the light of a diagnosis that explains things differently. It feels like it is important to understand what kind of expectations others have of you, what your strengths and weaknesses are, and what you can actually manage in relation to others. So that you don’t feel totally like a failure. That’s what I’ve always felt like; what a failure I think I am, which makes me feel completely depressed. (Alice)

The interviewees told how a diagnosis can work as a way of understanding oneself and as a source of identification or internal acceptance, where the diagnosis can provide them with support and the tools to manage challenges.

## Discussion

The young autistic adults in our study were involved in trying to understand their functionality and who they are, both at work and in their leisure time. The identified strategy used by them was to *know and accept oneself*. In other words, they were engaged in a process of developing their sense of self-awareness. This strategy was constituted by three substrategies: *learning from previous experiences*, *learning about oneself through securing the support of others* and *understanding and accepting autistic functionality*.

*Learning from previous experiences* involved the shaping of self-awareness through experience-based learning, whereby the young autistic adults learned about themselves by reflecting on previous experiences, such as trying to balance their use of energy between participation in activities and recuperation. The interviewees highlighted how their employers tend to not understand the consequences of their employees’ functionality, which results in unrealistic expectations of their work performance. The employer takes for granted that a person will understand things that, due to autistic functionality, can be difficult to grasp, or that they will perform in a neurotypical way (cf. Scott et al., 2019).

*Learning about oneself through securing the support of others* refers to how the support of significant others becomes a means of developing self-awareness through learning about both oneself and one’s functionality, and finding strategies to cope with challenging experiences (cf. Huang et al., 2017). During leisure time, these young adults choose to spend time with people who they want to socialize with and feel comfortable with. Spending time with people who share the same interests or with people who they are similar to helps to reduce stress since there is less need to adapt their behaviour to other people’s expectations. This informal support and opportunity to learn about oneself was often raised in connection with the transition to independent living and managing a household. Here, the relationship between private life and working life becomes clear – to manage your work you must be able to manage your private life, and vice versa.

The young adults compared themselves with others and wanted to fit in with societal norms that consider vocational work an essential part of everyday life (Cribb et al., 2019; First et al., 2019). Having moved to a place of their own as part of (a neuroconventional) adulthood, it is likely that the household chores increase while support from parents decreases. The participants rely on close and supportive relationships with their parents or partners to different degrees. For Hugo, invaluable informal support from his parents underlines the concept of ‘linked lives’, which highlights how events experienced by one family member have implications for the life course of another family member (see Hogan, 2012).

*Understanding and accepting autistic functionality* involves examining how self-awareness is constructed following a diagnosis. A diagnosis is a way of understanding oneself and a source of identification (internal acceptance), and provides support and tools for managing challenges (cf. Kiely et al., 2020; Lilley et al., 2021; Zener, 2019). A diagnosis might lead to increased acceptance of the informant’s needs by managers and colleagues in the workplace (external acceptance) and possibly even change non-autistic norms on how to work and be social at work. However, a diagnosis might also lead to stigmatization, where the autistic person is considered unable to carry out work, making autistic functionality the problem rather than focusing on transforming neuroconventional norms in the workplace (Crane et al., 2019; Lilley et al., 2021). To avoid stigmatization, some autistic people might use social camouflaging behaviours, in this case in order to pass as a non-autistic person in the workplace. This has been previously identified as a common ‘camouflaging context’ (Cage, Troxell-Whitman, 2019). Using Goffman’s (1986/1963) stigma theory, autism can be understood as a concealable stigma because it is not necessarily physically visible. Within Goffman’s framework, autistic people can be understood as ‘discreditable’, in that their stigma is not visible or can be hidden. However, according to Hugo, being discreditable or passing as non-autistic is not necessarily a positive experience. If his managers and colleagues disregard his difficulties and need for specific adaptations at his workplace, this can pose problems. Hugo may need to keep reminding his managers and colleagues that he is autistic in order to have his work adapted to his needs and functioning. In effect, this forces Hugo to continually ‘come out’ regarding his diagnosis and puts the responsibility for seeking adaptation on him (Cage & Troxell-Whitman, 2019).

Young autistic people often experience stigma, which can affect their mental health (Crane et al., 2019). It is therefore important to listen to and learn from young autistic people, understand their need for both internal and external acceptance, and challenge neuroconventional support and working life norms (Bertilsdotter Rosqvist, Hultman, et al., 2022; Bertilsdotter Rosqvist et al., 2023. In other words, instead of expecting autistic employees to adapt to non-autistic functionality, managers and autistic employees must cooperate to find ways to use strengths and provide support and adaptation in connection with potential workplace challenges (Bury et al., 2019; Scott et al., 2019; Webster & Garvis, 2020). Too much pressure risks overload, a state in which autistic people are overwhelmed with emotions and by perceptual input (cf. (Bury et al., 2019; Müller et al., 2008).

In conclusion, *self-awareness* is key to being able to understand and care for oneself. The young autistic adults in this study demonstrated how they are able to reflect on and develop their sense of self-awareness (Huggins et al., 2021; Lind et al., 2019; Sumiya et al., 2018). This calls into question research that posits autistic deficits in a sense of self-awareness (Kana et al., 2017; Mundy et al., 2010; Stout, 2019). Instead, we argue that autistic self-awareness – both how it may be developed among and how it can be expressed by autistic people – should not be contrasted with neurotypical development and expressions of self-awareness, but explored and nurtured on its own terms. The young autistic adults in our study have demonstrated how they are aware of and try to adapt to neuroconventional norms at the same time as they are developing and exploring self-awareness strategies and a self-awareness of their own (cf. Hobson, 2006; Lind & Bowler, 2009; Williams & Happé, 2010).

In the context of vocational work, self-awareness is required to recognize the situations in which stress occurs and, in relation to this, to be able to identify strategies that can alleviate stress and provide energy (cf. First et al., 2019; Mantzalas et al., 2022). When a person is aware of what he or she needs, it is easier to predict and plan for emotional well-being (cf. Cribb et al., 2019). It also becomes easier to ask for appropriate help and support.

### Limitations

These data are limited by the number of interviewees and interviews. Three of the four interviewees were men, and this might privilege autistic male perspectives. The interviewees are located in an urban context, which might affect the experiences of the interviewees compared, for example, to young autistic adults in more rural parts of Sweden or in other countries. However, these data are rich and detailed, and provide a nuanced understanding of the interviewees’ experiences of knowing and accepting oneself. Meeting the interviewer several times gave our participants an opportunity to further develop or clarify their perspectives from previous discussions on self-awareness and support needs.

### Implications for policy, practitioners and future research

We argue that for practitioners working with autistic individuals to both understand and support them, it is important to support their journey of self-awareness (Bertilsdotter Rosqvist, Hultman, et al., 2022; Bertilsdotter Rosqvist et al., 2023; First et al., 2019; South & Sunderland, 2022). Hence, practitioners must widen the scope of their support strategies by including both autistic people and professionals in the process of creating sustainable support interventions. Together, employers and support persons (both other autistic and non-autistic) can offer proactive and reactive support (Müller et al., 2008). It is therefore crucial to understand, include and support autistic employees’ journeys of self-awareness and their identified coping strategies. This could enable a shared decision-making process that includes the autistic employee and engages with important stakeholders, such as employers, co-workers, formal support persons and possibly family members and friends (Bertilsdotter Rosqvist, Hultman, et al., 2022; Bertilsdotter Rosqvist et al., 2023; First et al., 2019; South & Sunderland, 2022). We call for more research on autism and self-awareness that explores autistic people’s experiences and thoughts in diverse social contexts, including work.
